# Optimal Bond Constraint Topology for Molecular Dynamics Simulations of Cholesterol

**DOI:** 10.1021/acs.jctc.2c01032

**Published:** 2023-02-17

**Authors:** Balázs Fábián, Sebastian Thallmair, Gerhard Hummer

**Affiliations:** † Department of Theoretical Biophysics, Max Planck Institute of Biophysics, Max-von-Laue Straße 3, 60438 Frankfurt am Main, Germany; ‡ Frankfurt Institute for Advanced Studies, Ruth-Moufang-Straße 1, 60438 Frankfurt am Main, Germany; § Institute of Biophysics, Goethe University Frankfurt, 60438 Frankfurt am Main, Germany

## Abstract

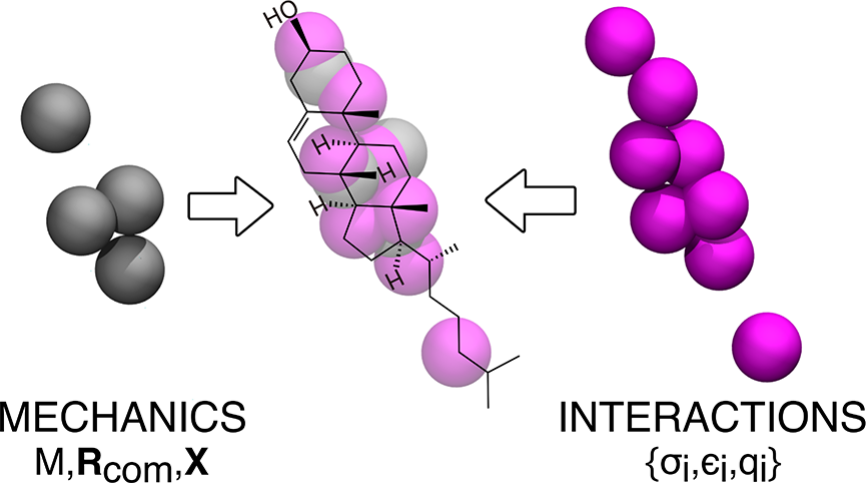

We recently observed artificial temperature gradients in molecular dynamics (MD) simulations of phase-separating ternary lipid mixtures using the Martini 2 force field. We traced this artifact to insufficiently converged bond length constraints with typical time steps and default settings for the linear constraint solver (LINCS). Here, we systematically optimize the constraint scaffold of cholesterol. With massive virtual sites in an equimomental arrangement, we accelerate bond constraint convergence while preserving the original cholesterol force field and dynamics. The optimized model does not induce nonphysical temperature gradients even at relaxed LINCS settings and is at least as fast as the original model at the strict LINCS settings required for proper thermal sampling. We provide a python script to diagnose possible problems with constraint convergence for other molecules and force fields. Equimomental constraint topology optimization can also be used to boost constraint convergence in atomistic MD simulations of molecular systems.

## 1 Introduction

Molecular dynamics (MD) simulations give us a molecularly detailed view of biological processes. To reach relevant length and time scales, the number of degrees of freedom can be reduced by coarse graining (CG). As one of the most successful approaches, the Martini force field^1,2^ on average maps four non-hydrogen atoms into a single bead. With the Martini force field, integration time steps of up to about Δ*t* = 30 fs can be used, compared to the 2 fs typical in all-atom simulations. CG force fields make it possible to study phase separation in lipid bilayers^3,4^ as possible models for the lipid rafts^5,6^ implicated in a myriad of cellular processes.^7−9^ However, we recently observed unphysical temperature gradients across the liquid-ordered (L_o_) and liquid-disordered (L_d_) phase boundaries in Martini 2 simulations of phase-separating ternary lipid mixtures.^10^ We traced these gradients to an insufficient convergence of the highly coupled bond-length constraints in the Martini 2 cholesterol model,^10,11^ with cholesterol being a major component of phase-separating lipid membranes. Insufficient constraint convergence also affects other observables at typical time steps, such as the diffusion coefficient or the contact fraction,^12^ which describes the degree of the phase separation in the system.

Slow convergence of the linear constraint solver (LINCS)^13,14^ is expected for any system with strongly coupled constraints. In Martini 2, this includes sterols such as cholesterol and ergosterol. In atomistic simulations, the issue arises, e.g., for angle-constrained butane or pentane, where LINCS fails entirely^13^ or cholesterol with all bonds constrained.^10^ Thus, an easy-to-use tool to estimate the required LINCS settings for proper convergence and a strategy to ameliorate bond length constraint issues would be valuable.

Here, we present a general method based on rigid-body mechanics^15^ and the use of virtual sites^16^ to optimize the LINCS convergence behavior of highly constrained topologies. Applied to the Martini 2 cholesterol topology, we obtain a cholesterol model that is optimized in terms of LINCS convergence but fully retains the original parametrization of the molecule in terms of force field and dynamics. First, we recapitulate the fundamental problem with the joint use of coupled constraints and the LINCS algorithm. We provide a set of guidelines that can help to avoid the creation of such constraints, and we present a script that enables the detection of constraint-related issues without explicitly performing any simulation. We apply the script to assess the behavior of constrained molecules in the Martini 3 small-molecule library^17^ and different atomistic cholesterol topologies. Second, we modify the cholesterol constraint topology to reduce the time-step dependence and the temperature gradients of the system. Finally, we perform Martini 2 simulations of (i) a single cholesterol molecule, (ii) a phase-separating lipid bilayer, and (iii) a G-protein-coupled receptor (GPCR) embedded in a cholesterol-containing membrane using both the original Martini 2 and our optimized cholesterol models to demonstrate the improvements introduced by our model.

## 2 Theory

### 2.1 LINCS Constraint Algorithm

As discussed in detail in the original LINCS papers,^13,14^ the intramolecular bond-length constraints are enforced after an unconstrained step according to
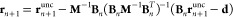
1where **r**_*n*+1_^unc^ is the position after the unconstrained time step *n* + 1, **M** is the diagonal matrix of particle masses, **B**_*n*_ is the gradient matrix of the constraint equations at time step *n*, and the vector **d** contains the prescribed length of the constraints. A key step in enforcing the constraints with eq 1 is the inversion of **B**_*n*_**M**^–1^**B**_*n*_^*T*^, a square matrix of dimensions *K* × *K* with *K* being the number of distance constraints. To reduce the computational cost, one rewrites the inverse in the form
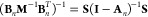
2where **S** is a diagonal matrix defined as the inverse square root^13^ of the diagonal of **B**_*n*_**M**^–1^**B**_*n*_^*T*^. Its elements are  with *i*_*A*_ and *i*_*B*_ indexing the two sites in distance constraint *i* = 1, ..., *K*. Note that eq 20 in ref (13) actually defines the inverse of **S**. **A**_*n*_ is a sparse symmetric matrix with zeros along its diagonal. The off-diagonal elements of **A**_*n*_ are given by the cosine of the angle between the constraints multiplied by a dimensionless mass factor. In LINCS,^13^ the inverse  is evaluated approximately in terms of a truncated geometric series
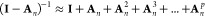
3This expansion is only applicable if the largest absolute value of the eigenvalues of **A**_*n*_ is less than one, , and it converges poorly as the magnitude of the eigenvalue approaches one. As the authors of LINCS note, angle-constrained butane has λ_max_ = 0.8 while angle-constrained pentane has λ_max_ = 1.2.^13^ The reason for coupled constraints being prone to fail is that increasing powers (*p*) of the **A**_*n*_ matrix represent the coupling effect of constraints that are *p* constraints away. The largest power *p* in the truncated series in eq 3 corresponds to the requested LINCS order (lincs_order). In coupled triangles, the third constraint away from a given constraint is already the constraint itself. As such, for highly coupled geometries, the expansion in eq 3 usually converges slowly or not at all. Consequently, the Gromacs MD simulation engine^18^ internally doubles the requested lincs_order for all constraints involved in triangular arrangements.^14^

As the **A**_*n*_ matrix can be diagonalized, one can conveniently assess the convergence of eq 3 with increasing lincs_order based on how fast λ_max_^*p*^ decreases. Under typical bond distortions, λ_max_ is approximately 0.4.^14^ Combined with lincs_order = 4, the error of the expansion is proportional to . Here, we use this relationship as a rule-of-thumb to estimate the lincs_order required for convergence.

### 2.2 Rigid-Body Mechanics

In Newton’s equations of motion, the mechanics of a rigid body is completely specified by the zeroth, first, and second moments of its mass distribution, namely, the total mass (*M*), the center of mass (CoM) vector (**R**), and the inertia tensor (**X**). Therefore, one is free to alter the positions of a set of rigidly connected, massive, noninteracting particles as long as *M*, **R**, and **X** are kept constant. Sets of points that possess the same *M*, **R**, and **X** are called equimomental systems.^15^ Naturally, to preserve the dynamics of the system, the massless interacting sites must subsequently be reconstructed around the new “scaffold” of massive noninteracting particles. This well-known concept is the basis of virtual sites in MD.^16^

While finding equimomental systems is trivial for rigid linear molecules, it becomes increasingly complicated for planar and nonplanar molecules due to couplings between *M*, **R**, and **X**. Recently, Laus and Selig presented a general procedure to generate equimomental systems from a regular tetrahedron.^15^ Here, we only present a brief outline of the theory. The central object of the formalism is the pseudo inertia tensor given by
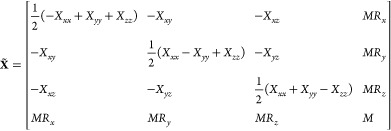
4The **X̃** matrix can be readily constructed from outer products of the homogeneous coordinates of the particle positions  as
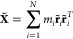
5where *m*_*i*_ are the masses of the individual particles. It is clear from eq 4 that setting the center of mass of a point cloud **R** = 0 and orienting it such that **X** is diagonal produces a pseudo inertia tensor  that is also diagonal. This transformation can be expressed as
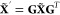
6where **G** is an element of the special Euclidean group SE(3) in the 4 × 4 matrix representation, and it encodes the translation and rotation of the point cloud of rigidly connected particles. Moreover, the individual terms in eq 5 are rank 1 matrices. Because four points not in a common plane already result in a full-rank matrix, any general rigid body can be replaced by a rigid structure with a minimum of four points.

Following ref (15) and without loss of generality, we assume in the following that we are in the principal frame of the rigid body, where by an appropriate translation and rotation the center of mass has been placed at the origin and the tensor of inertia is diagonal. The pseudo tensor of inertia is then also diagonal, . We define the diagonal matrix  and vectors  that satisfy . Then, **X̃** can be decomposed as
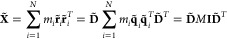
7where **I** is the 4 × 4 identity matrix. The last identity
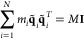
8follows from the fact that  by construction. Following ref (15), we now introduce a rotation in four dimensions, **U** ∈ SO(4), to define rotated 4-vectors . This 4D rotation leaves the pseudo inertia tensor unchanged
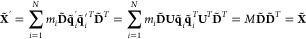
9where we used eq 8 and **UU**^*T*^ = **I**. The 4D rotations by **U** thus correspond to the required equimomental transformations.^15^

However, to interpret the 4D-rotated vectors  in terms of point masses in 3D, care must be taken to adjust also the masses *m*_*i*_ by mutiplying them with the square of the fourth element of the rotated 4-vector, . This ensures that  defines transformed 3D positions  with transformed masses *m*_*i*_^′^ that together leave **X̃** and thus the inertia tensor, center of mass, and total mass unchanged. Importantly, the resulting masses are bounded by 0 ≤ *m*_*i*_^′^ ≤ *M* with ∑_*i*_*m*_*i*_ = ∑_*i*_*m*_*i*_^′^ = *M*. In addition to the fact that 4D rotations allow one to create all possible equimomental systems, one has considerable freedom in fixing certain positions or masses of the final system.^15^ We note that in this process one can also change the number of mass points, e.g., by reducing the number of sites to the minimum of four for a general rigid body.^15^

## 3 Methods

### 3.1 Topology Optimization to Minimize λ_max_

We developed a python script using the MDAnalysis^19^ package to compute λ_max_ from a single molecular configuration. The use of a single configuration is justified only if the constrained particles do not undergo significant fluctuations during their motion. This condition holds for the Martini 2 cholesterol model as the two coupled triangles never substantially deviate from coplanarity. On the basis of the framework for the generation of equimomental systems^15^ introduced in the previous section, we minimized λ_max_ of **A**_*n*_ computed by the script with respect to equimomental configurations of a fixed number of rigid sites. In this way, we aimed at reducing the lincs_order required for properly constraining Martini 2 cholesterol.

We optimized the cholesterol model by minimizing the largest eigenvalue λ_max_ of the constraint matrix **A**_*n*_ as follows (see Figure S1 for illustration).

**Figure 1:**
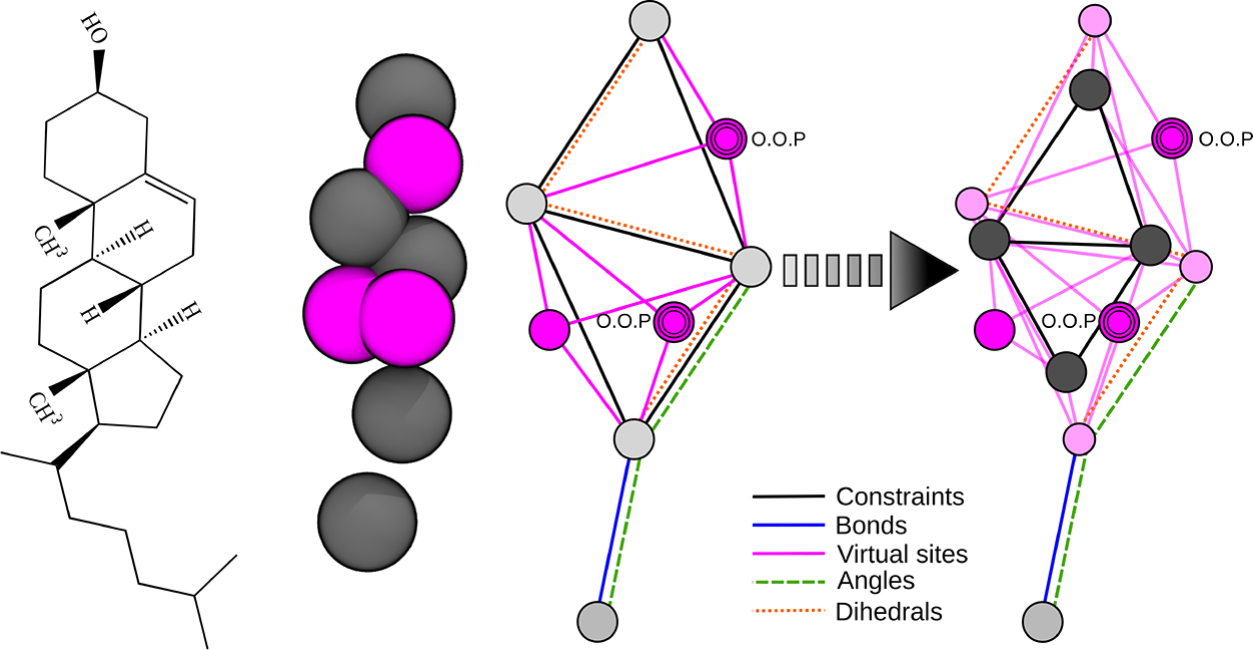
Constraint topology of the original and optimized cholesterol model in Martini 2. (Left) Structural formula. (Center) Martini 2 bead representation of the original cholesterol model rendered using VMD^28^ (gray, interacting beads carrying masses; magenta, virtual sites). (Right) Bond graph of the original and optimized models. Light and dark gray circles represent massive sites (with and without interactions, respectively), magenta circles are virtual sites in the original topology, and light pink circles are the newly introduced virtual sites (black lines, constrained bonds involving massive sites; magenta lines, constrained bonds between massive and virtual sites; blue line, flexible bond; green dashed line, flexible bond angle; red dotted line, flexible dihedral angle connecting the two constrained polyhedra, O.O.P, virtual sites out-of-plane with respect to the defining particles).

1. First, we decoupled the masses of the four beads involved in the two coupled constraint triangles from the interaction sites by introducing four additional noninteracting sites positioned initially at the location of the respective interacting bead. The cholesterol tail bead forming the fifth massive site was left unchanged throughout the optimization, being connected to the rest of the molecule by a flexible bond and thus not part of any constraint. The four newly introduced beads initially inherited the masses of the original beads, while the original beads became massless virtual interaction sites. As a result of the decoupling, the optimized model has 12 beads, 4 more than the original model.
2. Second, we iteratively optimized the positions and masses of the four newly introduced massive sites in an equimomental manner. We used eq 7 to find the matrix **D̃** and vectors  (*i* = 1, ..., 4) for the four massive sites. Then, at each iteration step, we generated random four-dimensional rotations **U** ∈ SO(4) for small rotation angles. The application of **D̃**U to  produced new homogeneous coordinate vectors  and hence provided new positions **r**_*i*_^′^ and masses *m*_*i*_^′^. Following Laus and Selig,^15^ this procedure guaranteed that *M*, **R**, and **X** remained fixed throughout the optimization process.
3. Then, we computed the **A**_*n*_ matrix of the newly generated configuration with modified positions and masses of the four massive constrained particles and determined λ_max_ and the estimate of the required lincs_order. Following a Monte Carlo scheme, we accepted any new configuration that lowered λ_max_ and repeated the refinement (steps 2 and 3) until a sufficiently low value was reached. To avoid pathological structures, we specified a minimum distance of 2.7 Å between any two beads as a constraint for the optimization.
4. After the iterative equimomental optimization of the masses and positions of the four newly introduced sites (black circles in Figure 1, right), the relative positions of the seven massless virtual sites (four originally massive interaction sites plus the three original virtual sites; shown as pink and magenta circles in Figure 1, right) were reconstructed in the reference frame of the four new sites.

### 3.2 MD Simulations

To compare the geometric properties of the original Martini 2 cholesterol model and our optimized model, we performed MD simulations of single isolated cholesterol molecules in the *NVT* ensemble using the two topologies. The simulations were 50 ns long each with time steps of 10 fs and lincs_order = 4. This combination of settings ensured sufficient convergence of the molecular properties.

We also performed MD simulations of ternary phase-separating lipid mixtures based on the simulations of Thallmair et al.^10^ Systems consisting of 1276 dipalmitoyl-phosphatidyl-choline (DPPC), 912 cholesterol, and 950 dilinoleoyl-phosphatidyl-choline (DLiPC) molecules corresponding to a molar ratio of 0.42/0.28/0.30 were built using the program *insane.py* in a random distribution.^20^ The bilayers were solvated in a 0.15 M NaCl solution. This resulted in an overall number of 38 917 CG water beads (10% of which were antifreeze particles) and 428 NaCl ion pairs. All simulations were performed with Gromacs^18^ version 2020.1. Energy minimization of the initial structures using a steepest descent algorithm was followed by two 500 ps pre-equilibration runs with time steps of 1 and 10 fs, respectively. The equilibration was concluded by a run of 1 μs, which is long enough for phase separation to occur. During production, we simulated 750 000 000 steps corresponding to 7.5 μs with Δ*t* = 10 fs, 15 μs with Δ*t* = 20 fs, and 22.5 μs with Δ*t* = 30 fs. The MD parameters and settings corresponded to the “New-RF” values.^21^

During the simulations, the pressure was maintained at 1 bar using a Parrinello–Rahman barostat^22^ with semi-isotropic coupling. We employed a coupling constant of τ_*p*_ = 12.0 ps and a compressibility of β = 3 × 10^–4^ bar^–1^. The temperature was kept constant at 310 K using a velocity rescaling thermostat^23^ with a coupling constant of τ_*T*_ = 1.0 ps. One thermostat was used for the solvent beads (water, antifreeze, and ion beads) and a second independent thermostat for the lipids.

We assessed the two different constraint topologies of cholesterol on the membrane properties by performing runs with the original Martini 2 cholesterol model and with the optimized geometry of virtual sites. We simulated both models using lincs_order = 4, 6, and 8 and time steps of Δ*t* = 10, 20, and 30 fs to check for possible temperature gradients and to evaluate the dependence of structural and dynamic properties on the lincs_order and the time step.

To investigate the differences in lipid–protein interactions, we performed simulations of a β_2_-adrenergic receptor (β_2_AR, PDB ID: 2RH1) embedded in an asymmetric lipid bilayer consisting of 65% POPC and 35% cholesterol in the upper leaflet and 55% POPC, 35% cholesterol, and 10% PIP_2_ in the lower leaflet. The initial configuration was obtained from Song et al.^24^ (personal communication) and simulated using lincs_order = 4, 6, and 8 and time steps of Δ*t* = 10, 20, and 30 fs to assess the impact of improper constraining on the interactions of β_2_AR and cholesterol. Three replicas of 15 μs length were simulated with each combination of lincs_order and time step, and the first 5 μs of every trajectory were discarded from analysis as equilibration. All reported quantities were averaged over the three replicas.

### 3.3 Analysis

To assess the achieved improvements in the optimized cholesterol model, we computed a range of observables for the original and optimized cholesterol models. To show that our approach does not alter the original model in any detectable fashion, we extensively compared the solvent-accessible surface area (SASA) and the equilibrium and RMSD values of pairwise bead distances of the single isolated cholesterols. The SASA was computed using gmx sasa and a probe sphere of radius 1.85 Å, while the bead distances were computed using a PLUMED 2.7^25^ script.

In the systems containing lipid bilayers, we computed the temperature of the different lipids using the Gromacs^18^ tool gmx traj. For the different cholesterol models, the temperatures calculated from the kinetic energies were corrected for the respective numbers of free and constrained degrees of freedom. The standard Martini 2 cholesterol consisting of eight beads has 3*N* = 24 degrees of freedom. However, the three massless virtual sites do not contribute to the kinetic energy. Together with the 5 constraints imposed on the structure, they leave only 10 degrees of freedom. Hence, a correction of 24/10 was applied to the temperature values. The same reasoning results in a correction factor of 36/10 for our optimized model.

To gain deeper insights into the temperature gradients of the phase-separating systems, we computed the lateral distribution of temperature in the membrane and its difference between DLiPC and DPPC lipids (Δ*T*) with an in-house Python script using the MDAnalysis library.^19^ The kinetic energy of the lipids was calculated through *E*_k_ = *mv*^2^/2, where *v* is the velocity and *m* is the mass of the particle. According to the equipartition theorem, the temperature was then obtained as , where *k*_B_ is Boltzmann’s constant and *N*_DoF_ is the number of actual degrees of freedom (corrected for the constraints). Finally, the temperature was binned into 2D histograms. For ease of representation, we averaged the 2D temperature maps along the axis parallel to the boundaries between the L_o_ and L_d_ regions, that is, between the low- and high-temperature domains.

The gmx mindist tool was used to compute the contact fraction between DPPC and DLiPC lipids defined as^12^
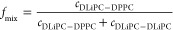
10where *c*_A–B_ is the number of contacts between species A and B within a cutoff radius of 0.7 nm. The contacts were evaluated based on the PO4 bead of the lipids.

The lateral diffusion coefficients of the phospholipid species were calculated from the mean square displacement of their CoM. The trajectories of individual lipids were unwrapped using the NPT-corrected scheme of von Bülow et al.^26^ and analyzed with a Generalized Least Squares estimator^27^ to obtain the diffusion coefficients. The lateral motion of the lipids was measured with respect to the CoM of the entire bilayer, thereby eliminating the drift of the overall CoM.

We analyzed the protein–lipid interaction between the β_2_-adrenergic receptor and cholesterol using the PyLipID package.^24^ Similar to the original paper, we used 0.475 and 0.80 nm for the dual cutoffs that deal with the “rattling effect” in lipid binding.^24^ The binding sites were required to consist of at least four residues. We computed the per-residue and per-binding-site lipid count, occupancy, duration, residence time, and unbinding rate *k*_off_ for each combination of lincs_order and time step, as in simulations of membranes without proteins. Additionally, we calculated the SASA and binding pose RMSD for the binding sites. The per-residue and per-binding-site observables computed by PyLipID were averaged over the 50 highest scoring residues and the top 3 scoring binding sites, respectively. See the original PyLipID paper^24^ for more details about the quantities.

## 4 Results and Discussion

### 4.1 Cholesterol Optimization Decreases λ_max_

In addition to three virtual sites, the Martini 2 model of cholesterol contains five massive sites, four of which form two coupled triangles. The original and optimized cholesterol models are illustrated in Figure 1.

Due to the presence of the coupled triangles, λ_max_ ≈ 0.95 and the estimated lincs_order is 72. Here and in the following, the internal doubling by Gromacs is not taken into account.^14^ The reason behind the high eigenvalues is closely related to the unequal masses and coupled, far-from-equilateral triangles involved in the constraints. While the use of equal masses and equilateral triangles would completely distort the topology of cholesterol, it would result in λ_max_ ≈ 0.50 and lincs_order = 5. The excessively large lincs_order of the original Martini 2 cholesterol is not only infeasible in simulations but would also be applied to all constraints in the system, not just the coupled ones with convergence issues.

Using the procedure outlined in the Methods section, we reduced λ_max_ from 0.95 to 0.80, corresponding to a decrease in the required lincs_order from 72 to 16. Even though the topology optimization described above efficiently reduced the required lincs_order, large distortions of the geometry of the original model can cause other instabilities. In the case of cholesterol, a further reduction in lincs_order was not possible, because the resulting topologies had massive beads that were too close. Due to the proximity of these massive beads, the integration of the equations of motion produced overly large deviations from the prescribed values, leading to a different kind of LINCS instability.

### 4.2 Optimized Model Leaves Cholesterol Geometry Intact

**Figure 2:**
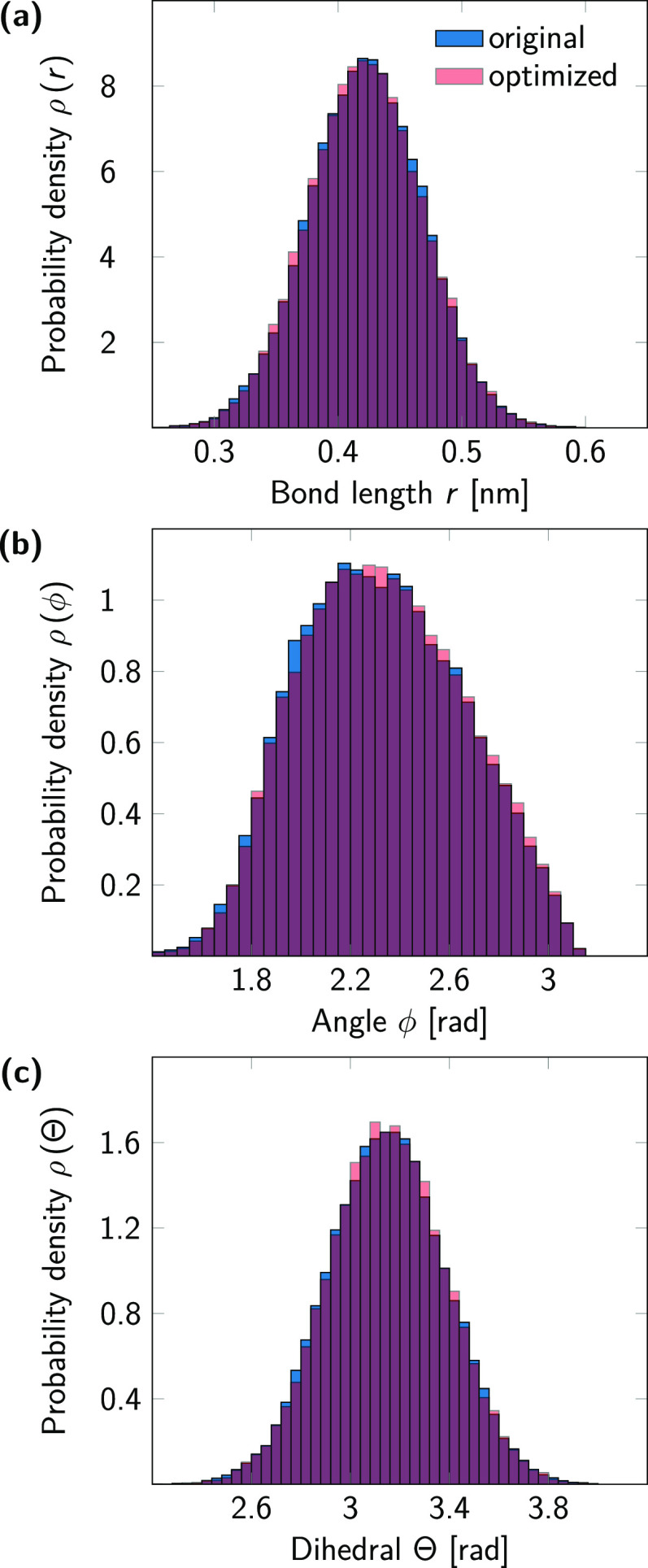
Bonded interactions in the original (blue) and optimized (red) cholesterol models. (a) Probability density function of the bond length *r*_C1–C2_. Bond lengths: 0.42 ± 0.05 nm (original), 0.42 ± 0.05 nm (optimized). (b) Probability density function of the angle ϕ_R3–C1–C2_. Angles: 2.32 ± 0.33 rad (original), 2.33 ± 0.33 rad (optimized). (c) Probability density function of the dihedral angle Θ_ROH–R2–R3–C1_. Dihedral angles: 3.15 ± 0.24 (original), 3.15 ± 0.24 (optimized).

We verified that the optimization procedure does not alter the configurations of the interacting beads by running simulations of a single cholesterol molecule in vacuum with both models. Our optimized model excellently reproduces the mean SASA value and its distribution (see Figure S2). To further prove the correctness of the optimized model, we computed all pairwise particle distances (Tables S1 and S2). The optimized model reproduces the mean values of all distances of the original model up to a precision of 0.02 Å as well as their standard deviation (compare Tables S3 and S4). Furthermore, we computed the probability density functions of the single bond, angle, and dihedral angle of the cholesterol models that do not rely on constraints or virtual sites. The distributions are virtually indistinguishable (Figure 2).

### 4.3 Optimized Model Eliminates Artificial Temperature Gradients in Phase-Separating Systems

**Figure 3:**
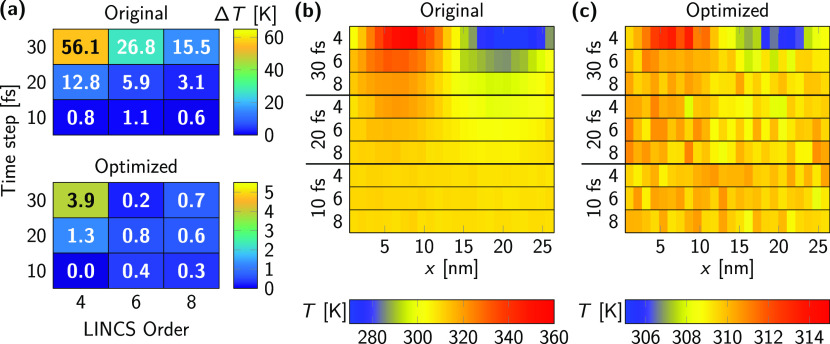
Temperature difference Δ*T* between DLiPC and DPPC lipids and between different phases in MD simulations of phase-separated bilayers using the original and optimized Martini 2 cholesterol model. (a) Δ*T* between DLiPC and DPPC lipids as a function of time step and lincs_order. We consistently set lincs_iter = 1. (b, c) Local temperature *T* along the *x* axis of the simulation box using the original (b) and optimized (c) Martini 2 cholesterol models. Note the different temperature scales in b and c.

We extensively compared the properties of lipid bilayers containing cholesterol described with the original and optimized model using a range of Δ*t* and lincs_order values. As a first test, we evaluated the average temperature difference between DLiPC and DPPC lipids, Δ*T* = *T*_DLiPC_ – *T*_DPPC_. For the original cholesterol model, the insufficient convergence of the LINCS algorithm led to the development of significant temperature differences, as shown in Figure 3. With the least strict LINCS settings (lincs_order = 4) and largest time step (30 fs), the differences can reach Δ*T* = 56 K between the two lipid types (Figure 3a). Decreasing the time step to 20 fs is not adequate even when lincs_order = 8 is used. To recover a temperature difference below 2 K with the original model, one has to use a 10 fs time step, incurring a significant penalty in the simulation performance.

The magnitude of the temperature gradient is even more striking when one examines the different membrane domains as a function of position (see Figure 3b and 3c). The reasons for the even larger temperature differences are that the individual phases are not composed uniquely of single lipid types and that the L_o_ phase contains the majority of cholesterol along with DPPC. We found a temperature difference between the two halves of the simulation box as high Δ*T* ≈ 80 K.

In contrast to the original cholesterol model, we observed only small temperature differences between the two phospholipid types with the optimized model. Even for a low lincs_order = 4 and long time step of 30 fs, the temperature difference is only 3.9 K (Figure 3a). For lincs_order = 6 and a 30 fs time step, Δ*T* drops to 0.2 K. Moreover, the temperature difference between the two membrane domains was in all cases under 8 K and became negligible for time steps below 30 fs or lincs_order = 8 for a 30 fs time step.

### 4.4 Properties of Phase-Separating System Converge When Temperature Gradients Are Eliminated

**Figure 4:**
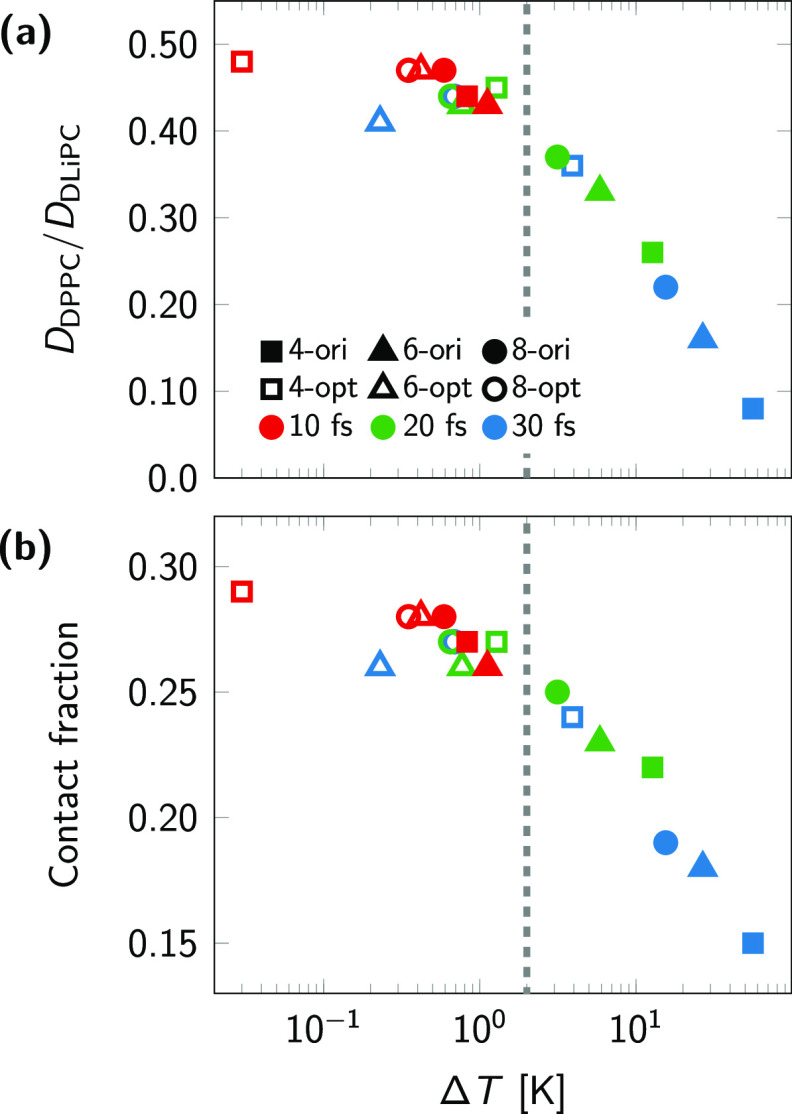
Effect of artificial temperature gradient in the membrane on its dynamic and static properties. (a) Ratio of the lateral diffusion coefficients of DPPC and DLiPC lipids. (b) Lipid–lipid contact fraction. Both quantities are shown as a function of the observed temperature difference Δ*T* of DLiPC and DPPC lipids in the respective systems. LINCS settings and time steps are indicated (see legend in a). Filled and empty symbols correspond to the original and optimized models, respectively, while colors red, green, and blue indicate the time step size. The vertical dashed line indicates the value of Δ*T* = 2 K, below which both observables appear to be Δ*T* independent within statistical uncertainties.

As a test of the dynamic properties, we computed the ratio of diffusion coefficients of DPPC and DLiPC lipids. As a probe of the local structure, we calculated the lipid–lipid contact fraction defined in eq 10. Results are shown as a function of the observed artificial temperature difference Δ*T* between DLiPC and DPPC lipids (Figure 4). Both quantities are greatly affected by Δ*T* and can only be considered converged within the sampling uncertainty when Δ*T* < 2 K (vertical dashed line). For the original cholesterol model, this requires a short time step of 10 fs; by contrast, for the optimized cholesterol model we have converged results in all cases except for the lowest lincs_order = 4 combined with the longest time step of 30 fs. Both models converge to the same values at small Δ*T*, further supporting the consistency of our optimization procedure.

**Figure 5:**
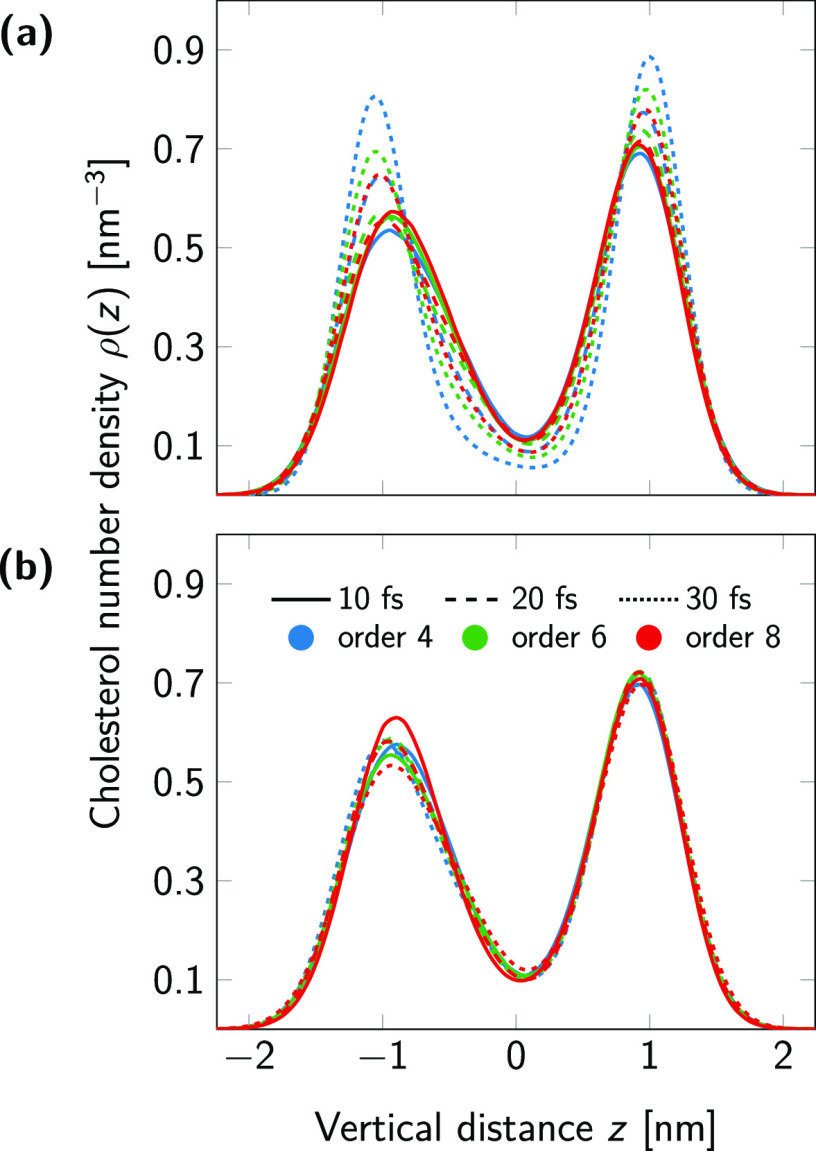
Number density of cholesterol (using the center of mass) along the membrane normal direction in the original (a) and optimized (b) models. Solid, dashed, and dotted lines correspond to the time step size. Blue, green, and red colors indicate lincs_order = 4, 6, and 8, respectively.

As another property impacted by temperature gradients, we computed the distribution of cholesterol along the membrane normal. Figure 5 indicates that while the cholesterol distribution is quite sensitive to the combination of time step and LINCS settings in the original model, all curves are on top of each other in the optimized model. Moreover, the original model converges to our optimized model in the limit of Δ*T* ≤ 2 K, that is, at small time steps and high lincs_order settings. The slight asymmetry in the width and height of the cholesterol populations of the two leaflets is due to the position restraints along the *z* axis of the DLiPC lipids in one of the two leaflets, which was applied to suppress membrane undulations.^29^

### 4.5 Cholesterol−β_2_AR Interactions Are Only Weakly Affected by Insufficient Constraining

**Figure 6:**
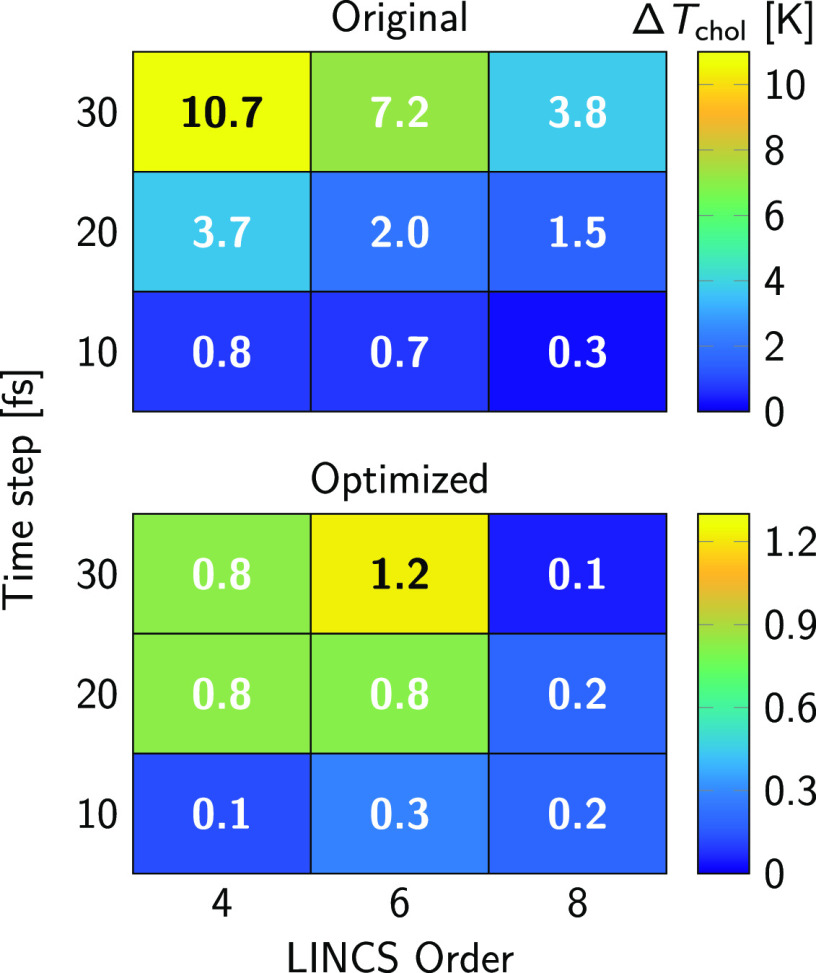
Temperature difference Δ*T*_chol_ between cholesterol and the thermostat target temperature (310 K) in simulations of β_2_AR-containing membranes using the original (top) and optimized Martini 2 cholesterol model (bottom) as a function of time step and lincs_order. Value of lincs_iter was in all cases kept equal to 1.

We investigated the interactions between cholesterol and a β_2_AR in a mixed-lipid, asymmetric bilayer, as described in the Methods section. Here, we analyzed the difference between the temperature of cholesterol and the reference temperature of the thermostat Δ*T*_chol_ = *T*_ref_ – *T*_chol_. The observed Δ*T*_chol_ values as a function of time step and lincs_order are shown in Figure 6 for the original (top) and optimized models (bottom). The large (>2 K) values of Δ*T*_chol_ indicate that the protein–lipid systems also suffer from the LINCS convergence issues observed in the phase-separating bilayers, albeit to a much lesser extent. This is due to a lower local cholesterol concentration compared to the L_o_ phase of the ternary system. By contrast, the systems simulated using the optimized model show virtually no temperature differences (see Figure 6 bottom) irrespective of the chosen parameters.

**Figure 7:**
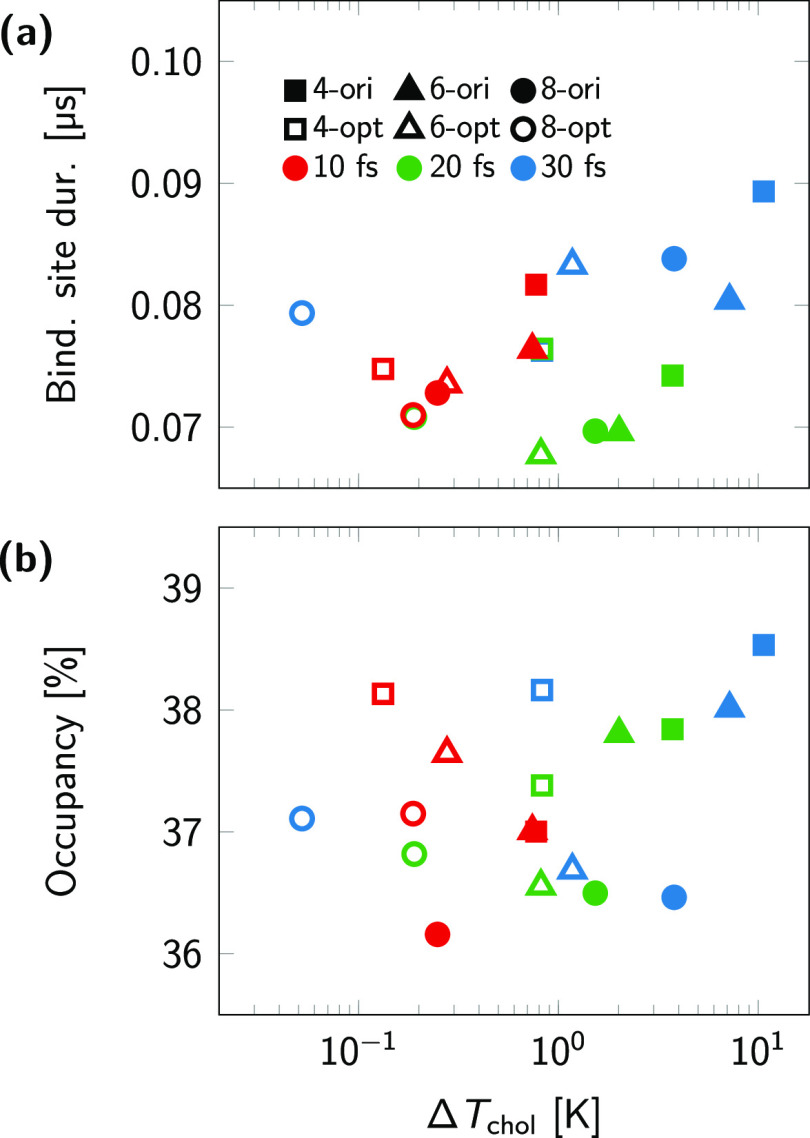
Duration of cholesterol contact (a) and occupancy (b) averaged over the top 3 binding sites and top 50 residues, respectively, in the β_2_AR-contaning membranes as a function of the temperature difference Δ*T*_chol_ between cholesterol and the thermostat target temperature (310 K). Filled and empty symbols correspond to the original and optimized models, respectively, while colors red, green, and blue indicate the time step size.

Despite the significant values of Δ*T*_chol_, the observables computed with PyLipID^24^ for the system containing β_2_AR do not exhibit systematic changes as a function of Δ*T*_chol_ in the case of the original model or significant differences between the two models (see Figures 7, S3, and S4). However, the system with the least strict settings (and largest Δ*T*_chol_) tends to be an outlier. Therefore, we discourage the use of lincs_order = 4 in combination with a long time step of Δ*t* = 30 fs in the investigated system. We also note that while there are no systematic differences between the two cholesterol topologies, the quantities determined using the optimized model have smaller variances (Figures S3 and S4). Moreover, if the lipid bilayers exhibit phase separation and thus have larger local cholesterol concentrations, we expect an increased impact of the nonconverged constraints on the protein–cholesterol interactions.

### 4.6 Optimal Cholesterol Model Improves Computational Performance

Results reporting on the computational efficiency of the original and the optimized cholesterol models in the phase-separating lipid bilayer are listed in Table 1. For details about the overall hardware configuration and the efficiency of the β_2_AR-containing simulations, we refer to section 6 of the Supporting Information and Table S5, respectively. For the given settings, MD simulations with the optimized cholesterol model incurred a performance penalty of ∼10–15% in all cases. Because this comparison does not take into account whether the physics of the system is correct or not, we also compared the performance of the previously recommended parameters for proper LINCS convergence (lincs_iter = 2, lincs_order = 12, Δ*t* = 20 fs) and (lincs_iter = 3, lincs_order = 12, Δ*t* = 30 fs).^10^ Requiring the temperature gradient to be negligible (|Δ*T*| < 2 K, based on the convergence of properties in Figure 4), one needs to perform simulations of the original model at Δ*t* = 20 fs using lincs_iter = 2, lincs_order = 12, resulting in 1623 ns/day, while it suffices to run the optimized model using lincs_iter = 1, lincs_order = 4, which allows the simulation of 1752 ns/day. The same is true at 30 fs, where one would have to run the original model with lincs_iter = 3, lincs_order = 12, giving 2264 ns/day. Our optimized model can run with lincs_iter = 1, lincs_order = 6 with a performance of 2453 ns/day, which represents a similar gain in performance. We conclude that MD simulations of the phase-separating bilayer model are at least as fast with the optimized cholesterol model as with the original model.

**Table 1:** Performance Comparison for MD Simulations of Phase-Separating Lipid Bilayers Using the Original and Optimized Cholesterol Models^a^

	lincs_order					
	original			optimized		
Δ*t* [fs]	4	6	8	4	6	8
30	2925	2801	2741	2608	2453	2403
20	2081	1887	1892	1752	1714	1664
10	1150	1076	1050	963	931	904

a Listed are simulated times in units of nanoseconds per day of wall-clock time. Results are shown as a function of time step Δ*t* and lincs_order at fixed lincs_iter=1.

Crucially, using our optimized model, other constrained molecules present in the simulation box are not subjected to the overly high LINCS requirements of the original cholesterol model. We evaluated the computational cost of unnecessarily constraining molecules in the simulations of the membrane-embedded β_2_AR using the same LINCS settings as for the protein-less phase-separating lipid bilayer. The protein model contains 462 constraints that do not require strict LINCS settings. The use of the optimized model results in an ∼30% performance increase compared to stricter LINCS settings^10^ (see Table S5).

### 4.7 Perspective on Other Potentially Affected Molecular Topologies

#### 4.7.1 Martini 3 Small-Molecule Library Does Not Suffer from LINCS Convergence Issues

The representation of a rigid topology by two connected triangular constraints, the so-called hinge model, as it is used in cholesterol, served as a blueprint for the Martini 3 topologies of a number of small molecules. Therefore, we assessed the quality of the constraint topology in terms of λ_max_ for 77 constrained Martini 3 small molecules^17^ (available at https://github.com/ricalessandri/Martini3-small-molecules) by estimating the required LINCS order for convergence and by performing explicit simulations (see Tables S6–S8). Our python script identified the molecules BZTA (benzothiazole), BZTH (benzothiophene), and MINDA (1-methylindazole) as having the largest eigenvalues of λ_max_ = 0.76. While these λ_max_ values are not excessively large, the reason behind them is the same as that for cholesterol: uneven masses and slightly distorted triangles. All three molecules were of planar, trapezoidal geometry with constraints applied to the four sides and the longer diagonal. As a test, we “flipped” the constraint along the diagonal of the molecules to constrain the other, shorter diagonal, which resulted in a decrease of λ_max_ in all three cases (BZTA and BZTH, 0.76–0.71; MINDA, 0.76–0.65).

In MD simulations using the *new-rf* input parameters^21^ and various combinations of lincs_order and time step, we found that all differences between the solute temperature and the thermostat reference temperature were less than 1.5 K and that the temperature difference between the solute and the solvent never exceeded 2 K (Tables S6–S8). Interestingly, “flipping” the diagonal constraint in the molecular topologies did not produce a clear improvement (Tables S6–S8), most likely due to the only moderately large λ_max_ values.

The explicit simulations fully support our conclusions drawn based on λ_max_. Remarkably, the eigenvalue analysis of all 77 molecules took less than 3 min on a standard laptop, while the explicit simulations require tests using various lincs_order and time step values and take a few hours per system using high-performance computers (running on a single node, 12 000 particles, and 15 million integration steps).

Finally, the topologies involve 2-to-1 mappings of non-hydrogen atoms to CG beads and contain “tiny” beads. The standard Martini 3 parameters of the “tiny” beads restrict the time step Δ*t* to well below 30 fs.^30^ While we did not encounter any crashes during the explicit simulations of the above systems, caution must be taken when other molecules with “tiny” beads are present.

#### 4.7.2 Atomistic Topologies with All Bonds Constrained Suffer from Poor Constraint Convergence in LINCS

In our previous study, we showed that atomistic systems containing cholesterol also can suffer from temperature gradients due to nonconverged constraints.^10^ Two examples are the CHARMM36 force field with hydrogen mass repartitioning (HMR)^31^ and the CHARMM36 model with hydrogens modeled as virtual sites (VIS).^32,33^ To allow time steps of up to Δ*t* = 5 fs, both models constrain all bonds. Although these cholesterol models do not contain any coupled triangles, larger rings of five or six atoms are present (Figure 1, left). Similar to three-membered rings, the resulting coupled constraints affect the convergence of the LINCS algorithm.

We analyzed the largest eigenvalues λ_max_ of the **A**_*n*_ matrix for the standard CHARMM36 cholesterol model as well as the HMR and VIS ones using our script. While the standard CHARMM36 cholesterol model is typically run by constraining solely bonds involving hydrogen atoms, HMR and VIS constrain all bonds to enable larger time steps. As expected, the standard CHARMM36 model^34^ has a low λ_max_ value of 0.06 because no coupled constraints are present. The other two models, however, exhibit considerably higher λ_max_ values of 0.73 (HMR) and 0.71 (VIS). For proper convergence, they would require lincs_order = 11 and 10, respectively. Note that the internal doubling of lincs_order is not initiated by Gromacs for these topologies because no constrained triangles are present.

This shows that also for atomistic systems in which all bonds are constrained, the analysis of the eigenvalues of the **A**_*n*_ matrix is a valuable diagnosis tool. Our script can be used to detect potential convergence issues of the LINCS algorithm and estimate the required LINCS settings.^18^

## 5 Conclusions

For phase-separating lipid bilayers, Martini 2 simulations with typical parameter settings have recently been found to suffer from substantial artificial temperature gradients across the phase boundaries. The locally different temperatures impacted other physical properties of the system such as the ratio of diffusion coefficients between the saturated and the unsaturated lipids, the degree of phase separation,^10,11^ and the distribution of cholesterol along the membrane normal direction (Figures 4 and 5). The origin of the artifact was traced back to insufficient convergence of the highly coupled bond constraints in cholesterol, one of the major components in such bilayers.

Here, we used the mechanics of rigid bodies^15^ to develop an optimization strategy for constraint molecular topologies to achieve quicker constraint convergence with the LINCS algorithm. We did not consider alternative ways of solving the constraint equations or other numerical methods to invert the matrix **I** – **A**_*n*_ in LINCS. In the optimization of the constraint topology, we minimized the largest absolute value of the eigenvalues of the **A**_*n*_ matrix, λ_max_. We also provide a python script to rapidly evaluate the quality of the constraint topology in terms of λ_max_ (available at https://github.com/bio-phys/constraint-coupling-analysis). We demonstrate the optimization strategy for the Martini 2 cholesterol model. By fully preserving the force field and the dynamics in the limit of infinitesimal time steps and perfect accounting for the constraints, the optimized model reproduces the single-molecule properties of the original model such as the solvent-accessible surface area or the bond/angle/dihedral distributions. With the exception of the largest time step and lowest lincs_order considered, the new model did not develop artificial temperature gradients in the phase-separating bilayer. The optimized model is publicly available at the Martini Web site (http://cgmartini.nl/images/parameters/ITP/martini_v2.0_CHOL_02-optLINCS.itp).

We further investigated the magnitude of the artifacts and their impact on cholesterol–protein interactions using a membrane-embedded β_2_-adrenergic receptor. Whereas the temperature of the original cholesterol model deviated significantly from the target temperature of the thermostat at larger time steps, there were no significant differences observed in lipid organization and dynamics around the protein between simulations with the original and the optimized cholesterol model. For MD simulations of membrane proteins with the optimized cholesterol model, we recommend the combined use of at least lincs_order = 6 with at most a 30 fs time step.

In the optimization of the cholesterol model, we ensure that the energetic and dynamic properties of the original model are fully maintained. The four additional beads in the constraint topology incur a computational cost. On the other hand, the stricter LINCS settings required for the original Martini 2 cholesterol model also impact the computational cost. In MD simulations of phase-separating ternary lipid mixtures with LINCS settings chosen to ensure similarly small temperature gradients, we achieved comparable performance with the original and optimized cholesterol model in terms of simulated time per wall-clock time (ns/day). The performance advantage of the optimized model increased to ∼30% in the presence of the membrane protein β_2_AR because the increase in the lincs_order required for the original cholesterol model applies also to constraints in the membrane protein.

We also analyzed the constraint topologies of the Martini 3 small-molecule library^17^ for the highest λ_max_, and we performed explicit simulations for the three molecules with the largest eigenvalues λ_max_ that corroborated the eigenvalue analysis. Overall, even for the largest λ_max_ = 0.76 we did not observe appreciable temperature gradients. The analysis and optimization method presented here can be readily incorporated into automatic topology builders and is potentially useful for other constrained molecules as well as rigid-body simulations.

We conclude by emphasizing the generality of the procedure described here to optimize the molecular constraint scaffold for rapid constraint convergence with LINCS. Possible applications include automated topology building of molecules,^35,36^ e.g., at the Martini 3 level of coarse graining.^35,36^

## Data Availability

Simulation input files and analysis scripts of this study are openly available on Zenodo at 10.5281/zenodo.7199702. The optimized topology is available at http://cgmartini.nl, while the python analysis script for the eigenvalues is available at https://github.com/bio-phys/constraint-coupling-analysis.

## Supporting Information Available

Solvent-accessible surface area of the original and optimized models, mean values and standard deviations of pairwise intramolecular distances of the original and optimized models, cholesterol−β2AR interactions computed by PyLipID, hardware configuration for the comparison of model performance, performance comparison of membrane-embedded β2AR simulations, and temperature differences in the Martini 3 small-molecule library (PDF)
