# Supplementary material for: Optimal Bond Constraint Topology for Molecular Dynamics Simulations of Cholesterol
Source: J Chem Theory Comput. 2023 Feb 17;19(5):1592–601. doi: 10.1021/acs.jctc.2c01032 (PMC10018735; doi:10.1021/acs.jctc.2c01032)
Supplement: Supplementary file 1 — ct2c01032_si_001.pdf [file ct2c01032_si_001.pdf]

Supplementary Information for: Optimal bond-constraint topology for molecular dynamics simulations of cholesterol

Balázs Fábián,*,† Sebastian Thallmair,‡ and Gerhard Hummer†,¶

†*Department of Theoretical Biophysics, Max Planck Institute of Biophysics, Max-von-Laue Straße 3, DE-60438, Frankfurt am Main, Germany*

‡*Frankfurt Institute for Advanced Studies, Ruth-Moufang-Straße 1, 60438 Frankfurt am Main, Germany*

¶*Institute of Biophysics, Goethe University Frankfurt, Frankfurt am Main, Germany*

E-mail: balazs.fabian@biophys.mpg.de

Contents

1 Main Steps of Optimizing the Cholesterol Topology **S3**

2 Solvent Accessible Surface Area of the two models **S4**

3 Mean values of pairwise particle distances in the two models **S5**

4 Standard deviations of pairwise particle distances in the two models **S6**

5 Cholesterol-β_2AR interactions computed by PyLipID **S7**

6 Hardware configuration for the comparison of model performances **S9**

7 Performance comparison of membrane-embedded β_2AR simulations **S10**

8 Temperature differences in the Martini 3 small molecule library **S11**

References **S13**

1 Main Steps of Optimizing the Cholesterol Topology

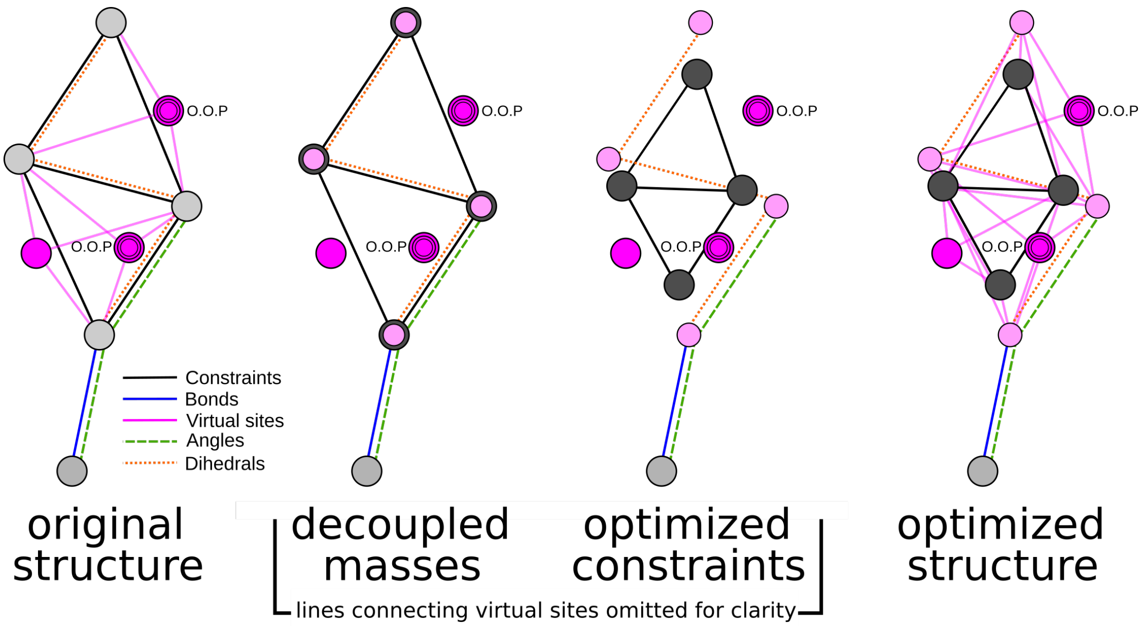

Figure S1: Main steps of optimizing the cholesterol topology as discussed in Section 3.1 of the main text. From left to right: the original cholesterol topology, decoupled masses (step #1 in the main text, optimized constraints for the non-interacting, massive sites (steps #2 and #3 in the main text), and the optimized structure with the original interacting sites reconstructed as mass-less virtual sites (step #4 in the main text). The light and dark gray circles represent massive sites (with and without interactions, respectively), the magenta circles are virtual sites in the original topology, and the light pink circles are the newly introduced virtual sites. Black lines: constrained bonds involving massive sites; magenta lines: constrained bonds between massive and virtual sites; blue line: flexible bond; green dashed line: flexible bond angle; red dotted line: flexible dihedral angle connecting the two constrained polyhedra, O.O.P : virtual sites out-of-plane with respect to the defining particles.

2 Solvent Accessible Surface Area of the two models

We compared the Solvent Accessible Surface Area (SASA) of the original and optimized models simulated with a single cholesterol in vacuum. The probability density function of SASA values is presented in Fig. S2. The SASA values were computed with `gmx sasa -ndots 4800 -probe 0.185`.

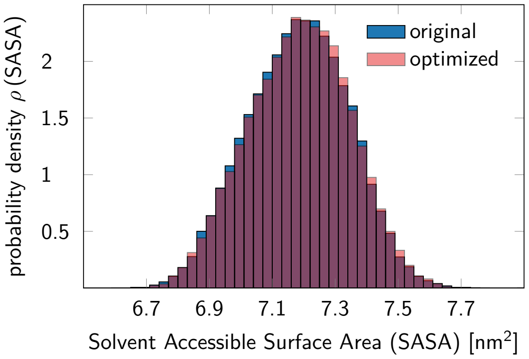

Figure S2: Solvent Accessible Surface Area of the original (blue) and optimized (red) cholesterol models. SASA values: 7.19 nm^2 ± 0.16 nm^2 (original), 7.18 nm^2 ± 0.16 nm^2 (optimized).

3 Mean values of pairwise particle distances in the two models

In Tables S1 and S2 we present the mean values corresponding to the pairwise particle distances in the original and the optimized model. The optimized model reproduces the mean values of all distances of the original model up to a precision of 0.02 Å, as well as their standard deviation (Tables S3 and S4).

Table S1: Mean values of all pairwise distances [Å] in the original cholesterol model, between all beads. The standard deviations of these distances are collected in Table S3.

	R1	R2	R2	R4	R5	C1	C2
ROH	2.83	4.90	6.02	8.13	7.97	11.34	14.34
R1		2.76	3.64	5.67	5.28	8.85	11.91
R2			2.72	3.46	4.05	7.24	10.21
R3				2.93	2.40	5.38	8.71
R4					2.18	4.05	7.04
R5						3.82	7.03
C1							4.23

Table S2: Mean values of all pairwise distances [Å] in the optimized cholesterol model, between all interacting beads. The standard deviations of these distances are collected in Table S4.

	R1	R2	R2	R4	R5	C1	C2
ROH	2.83	4.92	6.02	8.15	7.98	11.34	14.32
R1		2.77	3.64	5.69	5.28	8.85	11.93
R2			2.71	3.46	4.03	7.22	10.22
R3				2.94	2.40	5.38	8.73
R4					2.18	4.04	7.04
R5						3.81	7.05
C1							4.22

4 Standard deviations of pairwise particle distances in the two models

The standard deviations of the values in Tables S1 and S2 are collected in Tables S3 and S4 corresponding to a single cholesterol in vacuum. The values marked in blue indicate connections that are either constraints or a virtual site construction in the original model. Because all of the original particles are reconstructed using virtual sites in the optimized model, these connections are less rigid and possess somewhat larger standard deviations. The values in black are flexible in both models, and show practically identical standard deviations.

Table S3: Standard deviation of all pairwise distances [Å] in the original cholesterol model, between all beads. The mean values are in Table S1. The values marked in blue indicate connections that are either constraints or a virtual site construction in the original model.

	R1	R2	R2	R4	R5	C1	C2
ROH	0.00	0.00	0.00	0.06	0.20	0.07	0.88
R1		0.00	0.00	0.14	0.25	0.14	0.91
R2			0.00	0.00	0.00	0.00	0.97
R3				0.00	0.00	0.00	0.74
R4					0.00	0.00	0.99
R5						0.00	0.85
C1							0.46

Table S4: Standard deviation of all pairwise distances [Å] in the optimized cholesterol model, between all beads. The mean values are in Table S2. The values marked in blue indicate connections that are either constraints or a virtual site construction in the original model.

	R1	R2	R2	R4	R5	C1	C2
ROH	0.04	0.05	0.04	0.06	0.21	0.09	0.86
R1		0.05	0.04	0.14	0.25	0.14	0.91
R2			0.04	0.04	0.04	0.04	0.97
R3				0.04	0.04	0.04	0.74
R4					0.04	0.04	0.98
R5						0.04	0.85
C1							0.46

5 Cholesterol-β_2AR interactions computed by PyLipID

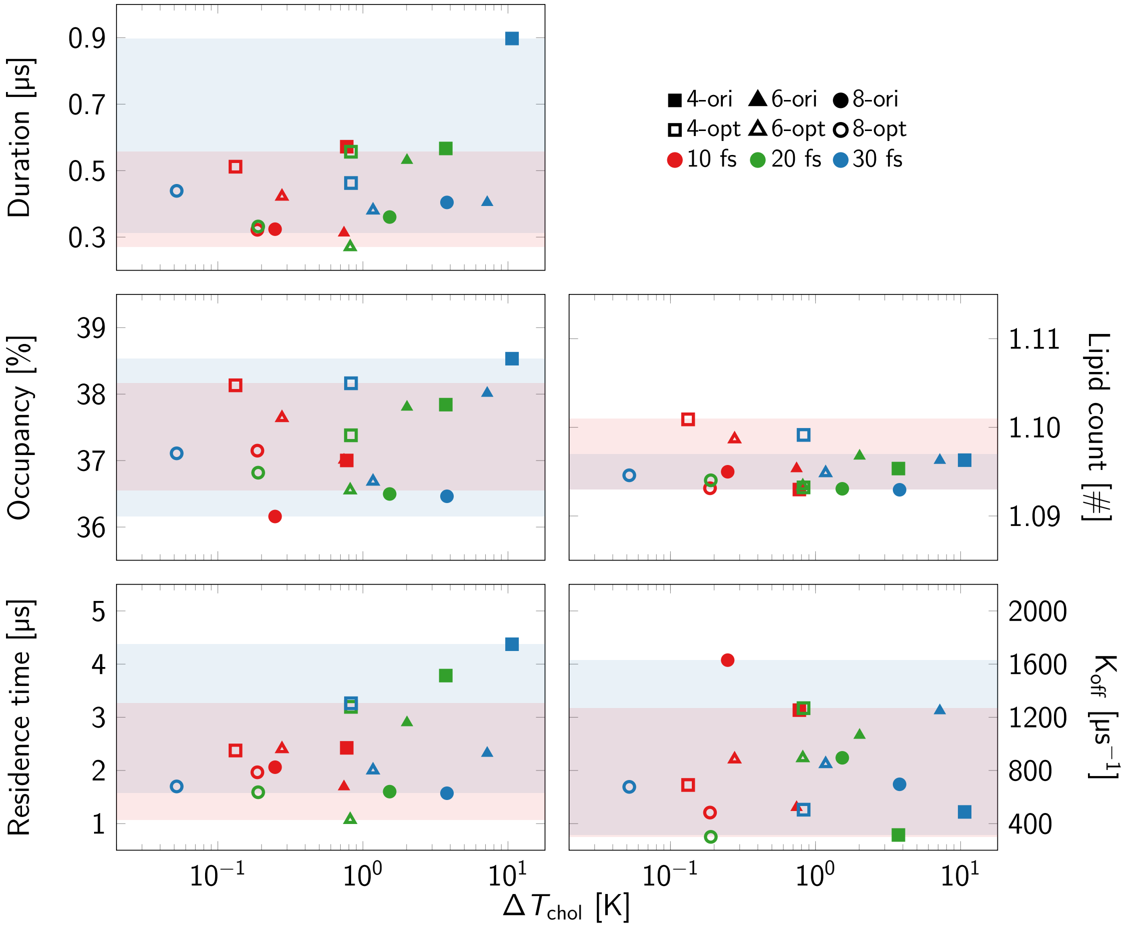

Figure S3: Quantities describing the residue-wise interactions of cholesterol and β_2AR as a function of ΔT_{chol} using various `lincs_order` (4,6,8) and time step (Δ_t = 10, 20, 30 fs). Solid and empty symbols correspond to the original and optimized models, respectively. The light blue and red regions are the range of values covered by the original and optimized models, respectively. All values have been averaged over the top 50 residues with the highest values.

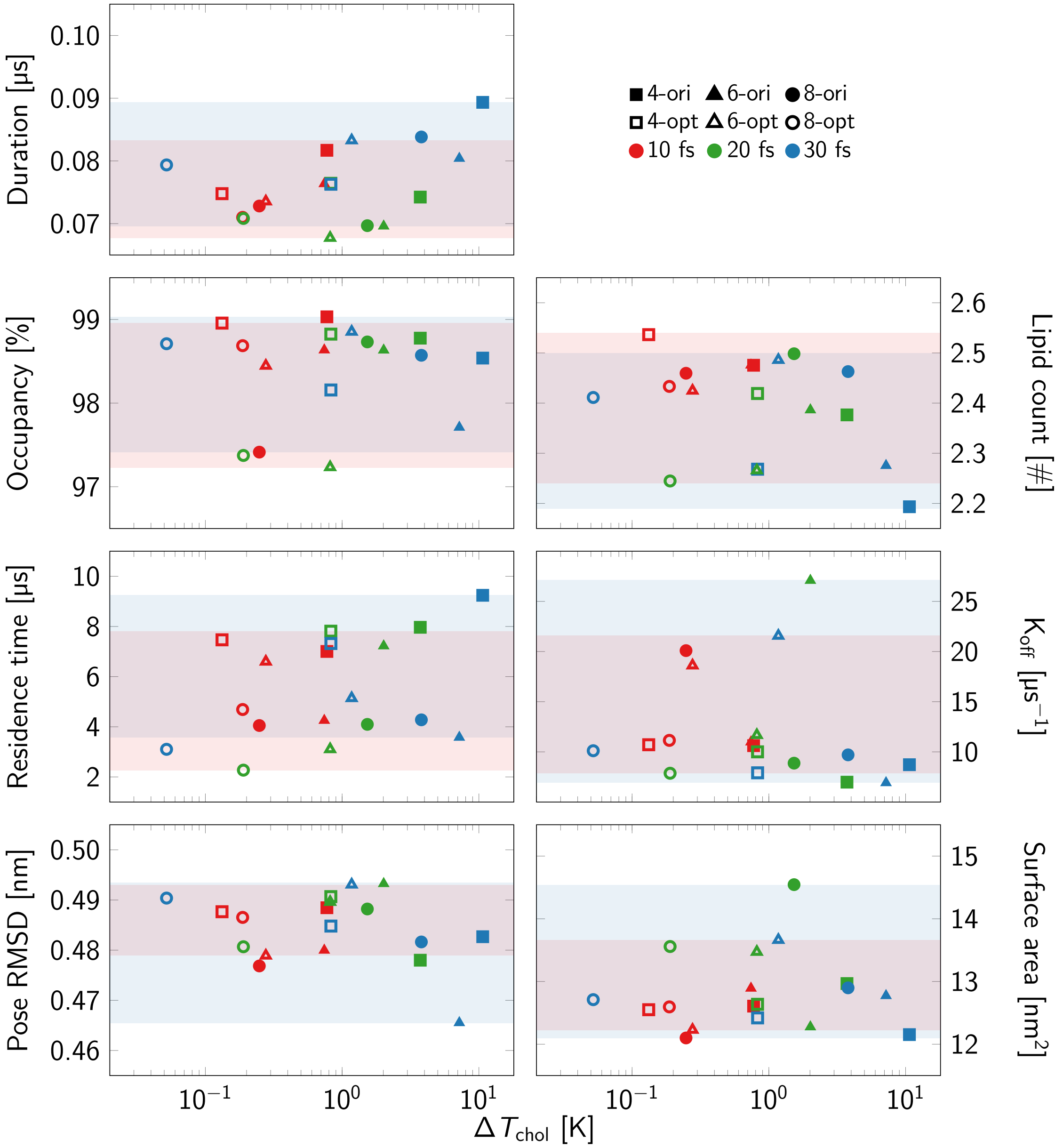

Figure S4: Quantities describing the binding-site-wise interactions of cholesterol and β_2AR as a function of ΔT_{chol} using various `lincs_order` (4,6,8) and time step ($\Delta_t = 10$, 20, 30 fs). Solid and empty symbols correspond to the original and optimized models, respectively. The light blue and red regions are the range of values covered by the original and optimized models, respectively. All values have been averaged over the top 3 binding sites with the highest values.

6 Hardware configuration for the comparison of model performances

```
GROMACS version:    2020.1
Precision:          single
Memory model:       64 bit
MPI library:        MPI
OpenMP support:     enabled (GMX_OPENMP_MAX_THREADS = 64)
GPU support:        CUDA
SIMD instructions:  AVX_512
FFT library:        fftw-3.3.8-sse2-avx-avx2-avx2_128-avx512
RDTSCP usage:       enabled
TNG support:        enabled
Hwloc support:      disabled
Tracing support:    disabled
CUDA driver:        11.40
CUDA runtime:       10.20

Running on 1 node with total 112 cores, 112 logical cores, 1 compatible GPU
Hardware detected on host ... (the node of MPI rank 0):
 CPU info:
  Brand:  Intel(R) Xeon(R) Platinum 8280 CPU @ 2.70GHz
 GPU info:
  #0: NVIDIA Quadro RTX 6000, compute cap.: 7.5, ECC: yes, stat: compatible
```

7 Performance comparison of membrane-embedded β_2AR simulations

Table S5: Performance comparison for MD simulations of membrane-embedded β_2AR using the original and optimized models. Listed are simulated times in units of nanoseconds per day of wall-clock time. Results are shown as a function of timestep Δt and `lincs_order` at fixed `lincs_iter`=1. Additional timings of the original model using the previously recommended settings[1] are 5210 ns/day (`lincs_iter`=2, `lincs_order`=12, Δt = 20 fs) and 6891 ns/day (`lincs_iter`=3, `lincs_order`=12, Δt = 30 fs).

	Original			Optimized		
	`lincs_order`					
Δt [fs]	4	6	8	4	6	8
30	10173	9989	9594	9765	9272	9149
20	6897	6904	6114	6737	6536	5976
10	3360	3814	3283	3723	3517	3085

8 Temperature differences in the Martini 3 small molecule library

We evaluated the temperature differences observed in systems containing the constrained molecules BZTA (benzothiazole), BZTH (benzothiophene) or MINDA (1-methylindazole) from the Martini 3 small molecules library[2] (https://github.com/ricalessandri/Martini3-small-molecules). These molecules were previously assessed to have relatively large $\lambda_{\max}$ (see the main manuscript). Eight replicas containing 1,728 of either molecule type were simulated in CG water (2,800 molecules) or octanol (950 molecules). The octanol simulations were repeated in four replicas using separate temperature coupling groups for the solute molecules and octanol. Finally, every molecule was tested using two topologies: the original Martini 3 version[2] and one where the constrained diagonal of the planar, trapezoidal molecule was "flipped" to act along the shorter diagonal instead of the longer one. All simulations were perfomed using the *new-rf* input parameters[3] and `lincs_order` = 4.

Table S6: The largest mean temperature differences $\Delta T_{298\mathrm{K}}$ (K, between the temperature of the molecule and the target temperature of the thermostat) and $\Delta T_{\mathrm{water}}$ (K, between the temperature of the molecule and the temperature water) observed across 8 replicas of the solute–water simulations. The apostrophe (') denotes the topologies using "flipped" constraints with lower $\lambda_{\max}$.

Water solvent, $\Delta T_{298\mathrm{K}}$						
	BZTA	BZTA'	BZTH	BZTH'	MINDA	MINDA'
10 fs	0.25	0.40	0.34	0.36	0.26	0.42
20 fs	0.88	0.45	0.62	0.78	0.54	0.53
30 fs	1.45	1.08	0.86	0.81	0.98	0.76
Water solvent, $\Delta T_{\mathrm{water}}$						
	BZTA	BZTA'	BZTH	BZTH'	MINDA	MINDA'
10 fs	0.44	0.59	0.40	0.52	0.33	0.51
20 fs	0.80	1.23	0.58	0.85	0.79	1.31
30 fs	1.97	1.69	1.48	1.83	1.51	1.86

Table S7: The largest mean temperature differences $\Delta T_{298\text{K}}$ (K, between the temperature of the molecule and the target temperature of the thermostat) and $\Delta T_{\text{octanol}}$ (K, between the temperature of the molecule and the temperature octanol) observed across 8 replicas of the solute–octanol simulations. The apostrophe (') denotes the topologies using "flipped" constraints with lower λ_{max}.

Octanol solvent, $\Delta T_{298\text{K}}$						
	BZTA	BZTA'	BZTH	BZTH'	MINDA	MINDA'
10 fs	0.17	0.30	0.34	0.30	0.24	0.32
20 fs	0.44	0.77	0.65	0.45	0.65	0.45
30 fs	0.99	0.55	1.35	0.72	0.82	0.47
Octanol solvent, $\Delta T_{\text{octanol}}$						
	BZTA	BZTA'	BZTH	BZTH'	MINDA	MINDA'
10 fs	0.66	0.45	0.47	0.32	0.33	0.44
20 fs	0.47	1.06	0.73	1.20	0.71	0.69
30 fs	0.86	1.15	1.77	1.32	1.25	0.62

Table S8: The largest mean temperature differences $\Delta T_{298\text{K}}$ (K, between the temperature of the molecule and the target temperature of the thermostat) and $\Delta T_{\text{octanol}}$ (K, between the temperature of the molecule and the temperature octanol) observed across 4 replicas of the solute–octanol simulations using separate temperature coupling groups. The apostrophe (') denotes the topologies using "flipped" constraints with lower λ_{max}.

Octanol solvent (separate coupling), $\Delta T_{298\text{K}}$						
	BZTA	BZTA'	BZTH	BZTH'	MINDA	MINDA'
10 fs	0.45	0.12	0.36	0.19	0.48	0.26
20 fs	0.28	0.41	0.38	0.28	0.51	0.49
30 fs	1.01	0.71	0.90	0.63	0.68	0.62
Octanol solvent (separate coupling), $\Delta T_{\text{octanol}}$						
	BZTA	BZTA'	BZTH	BZTH'	MINDA	MINDA'
10 fs	0.72	0.56	0.32	0.52	0.36	0.35
20 fs	0.59	0.71	0.16	0.49	0.58	0.77
30 fs	0.55	0.61	0.87	0.51	0.92	0.84

References

(1) Thallmair, S.; Javanainen, M.; Fábián, B.; Martinez-Seara, H.; Marrink, S. J. Nonconverged Constraints Cause Artificial Temperature Gradients in Lipid Bilayer Simulations. *The Journal of Physical Chemistry B* **2021**, *125*, 9537–9546.

(2) Alessandri, R.; Barnoud, J.; Gertsen, A. S.; Patmanidis, I.; de Vries, A. H.; Souza, P. C.; Marrink, S. J. Martini 3 Coarse-Grained Force Field: Small Molecules. *Advanced Theory and Simulations* **2022**, *5*, 2100391.

(3) De Jong, D. H.; Baoukina, S.; Ingólfsson, H. I.; Marrink, S. J. Martini straight: Boosting performance using a shorter cutoff and GPUs. *Computer Physics Communications* **2016**, *199*, 1–7.
